# Single-particle cryo-EM structural studies of the β_2_AR–Gs complex bound with a full agonist formoterol

**DOI:** 10.1038/s41421-020-0176-9

**Published:** 2020-07-07

**Authors:** Yanan Zhang, Fan Yang, Shenglong Ling, Pei Lv, Yingxin Zhou, Wei Fang, Wenjing Sun, Longhua Zhang, Pan Shi, Changlin Tian

**Affiliations:** 1 Hefei National Laboratory of Physical Sciences at Microscale and School of Life Sciences, University of Science and Technology of China, 230026 Hefei, Anhui China; 2 High Magnetic Field Laboratory, Chinese Academy of Sciences, 230030 Hefei, Anhui China

**Keywords:** Electron microscopy, Cell signalling

Dear Editor,

G-protein-coupled receptors (GPCRs) modulate cytoplasmic signaling in response to extracellular stimuli, and are important therapeutic targets in a wide range of diseases. Differential ligands binding to receptor promote different conformations of GPCR–G-protein complex, which can adopt diverse active states. Such ligand-directed biased agonism is now an important focus in drug discovery. Therefore, structure determination of GPCR–G-protein complexes in variable activation states is important to elucidate the mechanisms of signal transduction, and to facilitate drug discovery.

The β_2_-adrenergic receptor (β_2_AR), a canonical class A GPCR, is activated by adrenaline and norepinephrine^1,2^. Recent years, many agonists have been synthesized to stimulate the activation of β_2_AR, and some of these ligands have been developed for the clinical treatment of asthma and chronic obstructive pulmonary diseases^3^. Since the first crystal structure of β_2_AR bound with the inverse agonist carazolol was reported^4^, several crystal structures of the β_2_AR bound with different agonists have been determined. However, only structure of the BI167107-bound β_2_AR–Gs complex was determined to date, which represented the real active-state of β_2_AR^5^. Whether the observed β_2_AR–Gs interactions in the complex upon BI167107 binding provide a general rule for signal transductions from the binding of different agonists to cyclic adenosine monophosphate (cAMP) accumulation requires further validation, and also remains a major concern for the pharmacological understanding of β_2_AR and further drug development.

Formoterol is a selective, long-acting agonist of β_2_AR, which is unique as it both has a long-acting bronchodilator effect (> 12 h) and exhibits a fast onset of action (1–3 min from inhalation), suggesting that it is effective both as maintenance and reliever medication^6–8^. Herein, the cryo-EM structure of the formoterol-bound β_2_AR–Gs complex was determined with an overall resolution of 3.8 Å. Formoterol was reported to have a weaker affinity than BI167107 in β_2_AR binding, and also has lower β_2_AR activation potency than BI167107 (Fig. 1a). Therefore, comparisons between the structure of the formoterol–β_2_AR–Gs complex and the previously reported structure of the BI167107–β_2_AR–Gs complex will provide insights into the conformational responses of the β_2_AR upon binding to agonists with different potency.

**Fig. 1:**
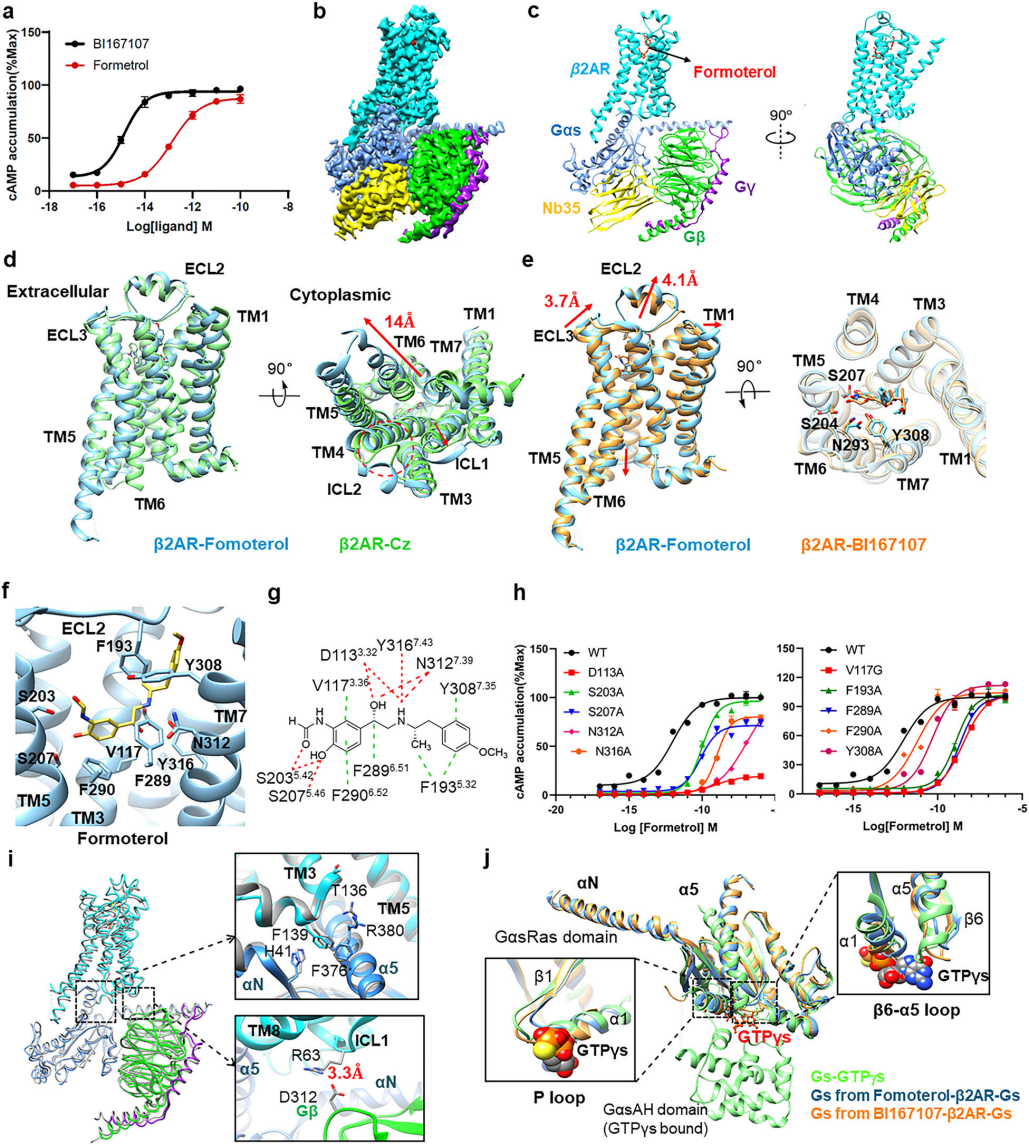
Cryo-EM structure of human β_2_AR–Gs complex bound with the agonist formoterol. **a** Agonist formoterol has lower activation potency on the β_2_AR than agonist BI167107. **b** Orthogonal view of cryo-EM density map of the formoterol–β_2_AR–Gs complex. Different colors are applied for β_2_AR (cyan), Gαs (blue), Gβ (green), Gγ (purple), and Nb35 (yellow). **c** Cartoon representation of structure of the β_2_AR–Gs complex, consisting of formoterol (red stick)-bound β_2_AR (cyan) and the Gs complex. **d** Cryo-EM structure of β_2_AR–formoterol (blue) was compared to the crystal structure of inverse agonist carazolol-bound β_2_AR-T4L (green). Cytoplasmic view of the superimposed structures showed significant structural changes. **e** Structural comparison between formoterol-bound β_2_AR (cyan) and BI167107-bound β_2_AR (orange). Notable differences are observed at the extracellular side of the receptor. Several residues involved in ligand coordination adopt different side chain conformations. **f** Side view of ligand-binding pocket in the formoterol-bound β_2_AR structure. Residues within 4 Å are shown in sticks. **g** Schematic representation of the interactions between β_2_AR and the ligand formoterol. **h** cAMP accumulation analysis of wild-type β_2_AR and mutants. Site mutations around the ligand-binding pocket disrupting the receptor-ligand interactions, resulting in β_2_AR malfunction in the cAMP accumulation assay. **i** Coupling interface between β_2_AR and Gs heterotrimer. In comparison with the BI167107–β_2_AR–Gs complex (gray), the residues (H41, F376 and R380 in Gs (blue), F139 in β_2_AR (cyan)) engaged in β_2_AR–Gs coupling in the formoterol–β_2_AR–Gs complex have notable structural changes. Direct interaction is observed between R63 in β_2_AR and D312 in the Gβ. **j** A comparison of the Gαs-Ras domain in the formoterol–β_2_AR–Gs complex (blue), BI167107–β_2_AR–Gs complex (orange) and Gαs–GTPγs (green). GTPγs is shown as balls and sticks. Both the P loop and the β6–α5 loop from the formoterol–β_2_AR–Gs complex (blue) stretched away from the guanine nucleotide-binding pocket, when compared with that in the BI167107–β_2_AR–Gs complex (orange) and Gαs–GTPγs (green).

First, we optimized the previously reported β_2_AR construct and obtained an engineered construct with improved expression in the sf9 insect expression system (Supplementary Fig. S1). The formoterol–β_2_AR–Gs complex in lauryl maltose neopentyl glycol (LMNG) detergent micelles was visualized using a Titan Krios microscope. After imaging and initial two-dimensional classification, three-dimensional classification yielded a final map at a global resolution of 3.8 Å (Fig. 1b; Supplementary Figs. S2, S3 and Table S1). The cryo-EM density map of the formoterol–β_2_AR–Gs complex exhibits well-resolved side chains, allowing rotamer placements for most amino acids (Fig. 1b; Supplementary Fig. S4). As revealed in Fig. 1c, the agonist formoterol is clearly identified in the orthosteric-binding site on the extracellular side of β_2_AR. The extensive receptor–Gs interface in the complex is mainly formed by the α5 helix in the Gαs-Ras domain, which extends into the transmembrane core of the receptor from the intracellular side. When compared the structure of formoterol-bound β_2_AR from cryo-EM complex with that of carazolol-bound β_2_AR in an inactive state (PDB: 2RH1), remarkable differences were observed for TM5, TM6 and ICL2 (Fig. 1d), suggesting that formoterol-bound β_2_AR is in an active-state.

When focusing on the structural details of the orthosteric-binding pocket, we found that the catecholamine phenoxy moiety of formoterol formed hydrogen bonds with Ser203^5.42^ and Ser207^5.46^ in TM5 (Fig. 1f, g; Supplementary Fig. S4). The alkylamine and the β-OH in the middle of formoterol formed polar interactions with Asp113^3.32^ in TM3 and with Asn312^7.39^ and Tyr 316^7.43^ in TM7. Moreover, formoterol formed hydrophobic interactions with receptors through V117^3.36^, F193^5.32^, F289^6.51^, F290^6.52^, and Y308^7.35^, stabilizing the orthosteric agonist-binding pocket in the active-state (Fig. 1g). cAMP accumulation assay revealed that mutation of the hydrophobic amino acids F193A, F289A, F290A, and Y308A in the formoterol-binding pocket decreased the potency of formoterol (Fig. 1h). Moreover, alanine substitution of residues D113, S203, S207, N312, and Y316 significantly impaired cAMP signaling (Fig. 1h). All of these results confirmed that residues involved in interactions between the ligand and β_2_AR play important roles in the formoterol-mediated cAMP signaling pathway.

When compared the cryo-EM structure of formoterol-bound β_2_AR with the crystal structure of BI167107-bound β_2_AR (PDB: 3SN6), significant differences were observed for extracellular regions, which contains the orthosteric ligand-binding pocket of the β_2_AR. Specifically, the extracellular top of TM1 extracellular region in formoterol-bound receptor moves outward by 3.2 Å when measured at the Cα carbon of Val34. ECL3, which connects TM6 and TM7, was also observed to extend slightly into the extracellular side (3.7 Å when measured at the Cα carbon of Asn301). Another notable difference observed between the two active-state β_2_AR structures was the short α-helix inside ECL2, which was observed to move upward by 4.1 Å when measured at the Cα carbon of Asn183 (Fig. 1e). It is worth noting that, when compared the crystal structure of BI167107-bound β_2_AR to the cryo-EM structure of BI167107-bound β_2_V_2_R (PDB: 6NI3), the ligand-binding pocket in the extracellular region is exactly the same (Supplementary Fig. S6). Thus, the structural differences observed between the cryo-EM structure of formoterol-bound β_2_AR and the crystal structure of BI167107-bound β_2_AR are not due to the steric restrains in the crystal lattice, but owing to the binding of different agonists. Taken together, these structural differences at the extracellular side of the receptors endow β_2_AR–formoterol with a slightly larger ligand-binding pocket. There are a total of ten amino acid residues that interact with formoterol in the orthosteric agonist-binding pocket, including five hydrophobic residues and five hydrophilic residues (Fig. 1g), compared with a total of 13 amino acid residues that interact with BI167107^5^ (Supplementary Fig. S5b). The decreased number of interacting residues between these two complexes might contribute to the lower affinity of formoterol versus that of BI167107^9^. Noteworthy, the side chains of both S204^5.43^ and N293^6.55^ rotate away from the formoterol molecule, which excludes the interactions stabilizing the binding between agonist and β_2_AR (Fig. 1e). Considering these observations, we speculate that the lower binding affinity of formoterol is mainly caused by the enlarged ligand-binding pocket and the reduced interactions between receptor and agonist due to changes of S204^5.43^ and N293^6.55^.

In the formoterol–β_2_AR–Gαs complex, the most extensive contacts between the G-protein and the β_2_AR are formed by the α5 helix of the Gαs-Ras domain, which inserts into the intracellular central cavity of the β_2_AR transmembrane domain, consequently leading to a 14 Å outward movement of TM6. Briefly, the interfaces are mediated mainly by extensive hydrophobic interactions (i) between the α5 helix of Gαs and ICL2, TM3, TM5, TM6 and TM7 of β_2_AR, and (ii) between the αN helix, αN–β1 loop of Gαs, and ICL2 of β_2_AR (Fig. 1i; Supplementary Fig. S7). As shown in Fig. 1i, the imidazole ring of H41 in the αN helix and the phenyl ring of F376 in the α5 helix from Gαs protein in the formoterol–β_2_AR–Gs complex rotate away from the hydrophobic pocket compared with that in the BI167107–β_2_AR–Gs complex, which might attenuate the hydrophobic interactions between the αN helix, αN/β1 loop of Gαs and ICL2 of β_2_AR (Fig. 1i). Since the hydrophobic pocket between β_2_AR and Gαs protein is crucial for GDP release and is probably necessary for the stabilization of the nucleotide-free β_2_AR–Gs complex, the decreased hydrophobic interaction in the formoterol-bound β_2_AR–Gs structure might have an impact on subsequent signal transduction^5^. Moreover, the side chain of R380 in Gαs protein from the formoterol–β_2_AR–Gs complex has a notable rotation away from TM3 relative to that in the BI167107–β_2_AR–Gs complex. The side chain rotation increases the distance between R380 in Gαs protein and T136 in β_2_AR, hence making it impossible to maintain the corresponding polar interaction found in the BI167107–β_2_AR–Gs complex.

A new interface absent in the structure of the BI167107–β_2_AR–Gs complex was observed between the Gβ protein and ICL1 of β_2_AR, which is mediated by the charge interaction between residue R63^ECL1^ in β_2_AR and residue D312 in the Gβ protein (Fig. 1i). To be noted, a similar interface was observed in the interaction between Gβ and class F GPCR^10^ or between Gβ and helix 8 of the class B GPCR^11,12^. Taken together, in comparison to the structure of BI167107-bound β_2_AR–Gs, the attenuated hydrophobic interaction between αN–β1 loop of Gα and ICL2 of the receptor, combined with the disappeared polar interaction between T136 in TM3 and R380 in α5 helix, might decrease the coupling interaction between β_2_AR and the Gα-Ras domain. This is consistent with the observed lower G-protein activation potency of formeterol versus BI167107 (Fig. 1a). Thus, structural comparison between the formoterol- and BI167107-bound β_2_AR–Gs complexes provides insights into conformational differences that are responsible for the distinct cAMP accumulation potency of different agonists.

Owing to the intrinsic flexibility, the density of the α-helical domain (αHD) could not be well-resolved, and the αHD was, therefore, excluded from the high-resolution map of the formoterol–β_2_AR–Gs complex. Superposition of the three Gαs-Ras domains from our cryo-EM structure of the formoterol–β_2_AR–Gs complex, a previously reported crystal structure of the BI167107–β_2_AR–Gs complex and the crystal structure of the Gαs-GTPγS complex (PDB:1AZT)^13^ revealed pronounced conformational differences for the α5 helix, which displaced toward the receptor in the two agonist-bound β_2_AR–Gs complexes versus that in the Gαs–GTPγS complex (Fig. 1j). In Gαs proteins, β6–α5 loop and β1–α1 loop (P loop) in the Gαs-Ras domain were reported to interact directly with the guanine ring and the diphosphate of nucleotide^14^. As nucleotide exchange is the essential step in cAMP accumulation during the signal transduction of the activated GPCR, conformational changes of these loop regions will directly affect the potency of GPCR. As shown in Fig. 1j, both P loop and β6−α5 loop in formoterol–β_2_AR–Gs displaced outward from the nucleotide-binding site, when compared with those of BI167107–β_2_AR–Gs. We suggest that the displacement of the P loop and β6−α5 loop from the nucleotide-binding site may attenuate their interaction with the guanine ring and diphosphate in GTP, further decreasing the catalytic efficacy of Gαs-Ras toward GTP. This might in turn be responsible for the observed lower potency of β_2_AR binding to formoterol than that to BI167107 (Fig. 1a).

In summary, here we report the cryo-EM structure of β_2_AR–Gs complexed with the high-affinity full agonist formoterol. When compared with the BI167107-bound β_2_AR–Gs complex, structural differences were observed at the extracellular side of the receptors, which endow formoterol-bound β_2_AR with a slightly larger ligand-binding pocket. Besides, the side chains of S204^5.43^ and N293^6.55^ in formoterol-bound β_2_AR rotate away from the ligand-binding pocket, which reduces the interaction between formoterol and β_2_AR. We suggest that these structural differences might be responsible for different affinities and activation potency of agonists formoterol and BI167107, and thus residues involved in these structural differences might be potential targets for new agonist design and drug development. Moreover, the influence of attenuated interactions between the Gαs-Ras domain and β_2_AR will be transduced to the nucleotide-binding pocket, ultimately leading to a lower GTP-binding affinity and hydrolytic activity of Gαs. The decreased interactions between the Gαs-Ras domain and β_2_AR observed in our structure of the formoterol–β_2_AR–Gs complex might in turn be partially responsible for the lower affinity of β_2_AR for formoterol, when compared with that of BI167107–β_2_AR–Gs complex structure^5^. These findings enrich our understanding of ligand-binding interactions and cAMP accumulation potency, enabling the exploration of new avenues for the development of innovative drugs targeting β_2_AR.

Density maps and structure coordinates have been deposited to the Electron Microscopy Database and the Protein Data Bank with accession numbers EMD-30249 and 7BZ2.

## Supplementary information

Supplementary Information

## Supplementary information

**Supplementary Information** accompanies the paper at (10.1038/s41421-020-0176-9).
