# Supplementary material for: Single-particle cryo-EM structural studies of the β2AR–Gs complex bound with a full agonist formoterol
Source: Cell Discov. 2020 Jul 7;6:45. doi: 10.1038/s41421-020-0176-9 (PMC7338445; doi:10.1038/s41421-020-0176-9)
Supplement: Supplementary file 1 — Supplementary Information [file 41421_2020_176_MOESM1_ESM.pdf]

Supplementary Materials for

Single-particle cryo-EM structural studies of β_2AR-Gs complex bound with full agonist formoterol

Yanan Zhang[1], Fan Yang[1], Shenglong Ling[1], Pei Lv[1], Yingxin Zhou[1], Wei Fang[1], Wenjing Sun[1], Longhua Zhang[1], Pan Shi[1], Changlin Tian[1,2]

[1]Hefei National Laboratory of Physical Sciences at Microscale and School of Life Sciences, University of Science and Technology of China, Hefei 230026, China

[2]High Magnetic Field Laboratory, Chinese Academy of Sciences, Hefei, 230030, China

These authors contributed equally: Yanan Zhang, Fan Yang, Shenglong Ling

Correspondence: Shenglong Ling (lsl@ustc.edu.cn)

Pan Shi (shipan@ustc.edu.cn), or Changlin Tian (cltian@ustc.edu.cn)

Materials and Methods

Expression and purification of human β_2AR.

The human β_2AR was truncated[1] at C-terminal from residue 348. N-terminal FLAG tag and C-terminal 10 × His tag was added for purification. This construct was cloned into pFastBac1 vector and expressed in Sf9 insect cells using the Bac-to-Bac Baculovirus system. The cells at a density of 2.5×10^6 cells per ml were infected with baculovirus at 27 ℃ for 60 h.

Cells were resuspended and grinded sufficiently in low salt buffer (10 mM HEPES, pH 7.5, 10 mM $MgCl_2$, 20 mM KCl, protease inhibitors named cocktail (Sigma, S8820)), then ultracentrifuged at 180,000g for 45 min. Precipitate were resuspended and grinded with high salt

buffer (10 mM HEPES, pH7.5, 10 mM $MgCl_2$, 20 mM KCl, 1M NaCl, protease inhibitor cocktail). Purified membranes were resuspended in storing buffer (10 mM HEPES, pH7.5, 10 mM $MgCl_2$, 20 mM KCl, 30% glycerol, protease inhibitor cocktail). Then, 10 μM formoterol were added, incubating 1 h at 4 ℃. Same volume of dissolution buffer (10 mM HEPES, pH 7.5, 1% DDM, 0.2% CHS, 1 M NaCl) were mixed with membrane solution and incubated 2 h at 4 ℃. After centrifugating at 180,000 g for 45 min, supernatants were pooled and incubated with preprocessed TALON Metal Affinity Resin (Clontech) overnight at 4 ℃. The resin was collected and washed with washing buffer 1 (50 mM HEPES, PH7.5, 0.05% DDM, 0.01% CHS, 500 mM NaCl, 20 mM IMD , 10 μM formoterol, 10 mM $MgCl_2$), washing buffer 2 (25 mM HEPES, PH7.5, 0.05% DDM, 0.01% CHS, 500 mM NaCl, 30 mM IMD, 10 μM formoterol), and finally eluted in buffer consisting of 50 mM HEPES, PH 7.5, 0.01% DDM, 0.002% CHS, 150 mM NaCl, 300 mM IMD, 10 μM formoterol. The eluted protein was concentrated and further purified by size-exclusion chromatography on Superdex 200 10/300 GL column (GE Healthcare) in 20 mM HEPES pH 7.5, 150 mM NaCl, 5 mM EDTA, 0.01% (w/v) DDM and 0.002% (w/v) CHS.

Expression, purification of Gαs, Gβγ and formation of Gs complex.

The human Gαs was cloned into pET28a vector. The contruct was expressed in *E.Coli* (BL_{21}(Gold)). When bacteria was cultured to a density of OD_{600}=0.8 at 37 ℃ in LB medium containing 100 mg/mL kanamycin, a final concentration of 0.8 mM isopropyl-β-Dthiogalactopyranoside (IPTG) was added into medium. Cells were then cultured at 20 ℃ overnight before harvest. The bovine Gβ and Gγ were expressed in Sf9 insect cells using

the Bac-to-Bac Baculovirus system. The cells at a density of 2.5×10^6 cells per ml were infected with baculovirus at 27 ℃ for 60 h.

Gαs and Gβγ have similar purification steps. Cells were harvested and resuspended with buffer (50 mM HEPES, pH7.5, 200 mM NaCl, 1 mM MgCl and 5.6 mM beta-mercaptoethanol (β-ME) and lysed by high pressure homogenizer. Cell debris were removed by centrifugation at 14000 rpm for 30 min at 4 ℃. The over-expressed proteins were purified using Ni-NTA affinity column (QIAGEN) and eluted with buffer (50 mM HEPES, pH7.5, 200 mM NaCl, 250 mM imidazole, 5.6 mM β-ME). Then, the target proteins were concentrated and further purified on Superdex 200 10/300 GL column equilibrated with buffer (50 mM HEPES, pH 7.5, 200 mM NaCl, and 5.6 mM β-ME). To form the Gs complex, Gαs and Gβγ were mixed together at molar ratio 1:1 and then incubated at room temperature for 1 h. Gαsβγ complex was isolated on a Superdex 200 10/300 GL column (GE Healthcare) with buffer 20mM HEPES, pH=7.5, 100mM NaCl, 1mM MgCl2, 1mM EDTA, 100uM TCEP.

Expression and purification of Nb35.

Nanobody-35 (Nb35)[2] was cloned into pET22b vector and expressed in *E.Coli (BL21(Gold))*. Bacteria were grown at 37 ℃ in LB medium containing 100mg/mL ampicillin to a density of OD_{600} = 0.8, a final concentration of 0.8 mM isopropyl-β-Dthiogalactopyranoside (IPTG) was added into medium. Cells were then cultured at 20 ℃ overnight. The bacteria were harvested with buffer consisting of 50 mM HEPES, pH7.5, 200 mM NaCl, 1 mM MgCl and 5.6 mM β-ME, and then lysed by high pressure homogenizer. Cell debris were removed by centrifugation at 14000 rpm for 30 min at 4 ℃. The over-expressed proteins were purified using Ni-NTA affinity column and eluted with buffer consisting of 50 mM HEPES, pH7.5, 200 mM NaCl, 250 mM

IMD, 5.6 mM β-ME. The eluted protein was concentrated and further purified by size-exclusion chromatography on Superdex 200 10/300 GL column (GE Healthcare) in 50 mM HEPES, pH7.5, 200 mM NaCl, 5.6 mM β-ME.

β_2AR-Gαs complex preparation

The purified β_2AR and Gs complex were mixed at a molar ratio 1:1.2 in buffer 1 (20 mM HEPES, pH7.5, 100 mM NaCl, 0.1% DDM, 1 mM EDTA, 3mM MgCl2, 10 μM formoterol) and incubating at room temperature. To maintain the high-affinity nucleotide-free state of the complex, 25 mU/mL Apyrase was added to hydrolyse GDP from Gαs binding pocket. To further maintain the stability of complex, Nb35 was added into the mixture and then incubating for 1 h at room temperature. The formoterol-bound β_2AR-Gαs complex was concentrated and further purified by size-exclusion chromatography on Superdex 200 10/300 GL column (GE Healthcare) in buffer consisting of 20 mM HEPES, pH7.5, 100 mM NaCl, 0.02% LMNG, 10 μM formoterol.

Functional analysis by cAMP assay

The function data of β_2AR was measured based on intracellular cAMP assay using cAMP-Gs dynamic kit[3] (Cisbio). HEK293T cells were cultured in DMEM medium supplemented with 10% (v/v) fetal bovine serum, 50 μg/mL penicillin and 50 μg/mL streptomycin using 37 ℃ incubator supplied with 5% CO_2. Cells were seeded onto 6-well cell culture plates before transfection. After overnight culture, the cells were transfected with β_2AR-pcDNA3.1 plasmid (or β_2AR mutation plasmid) using Lipofectamine 3000 transfection reagent (Invitrogen). About 12 h after transfection, the cells were collected, and suspended in DMEM containing 500 μM 3-isobutyl-1-methylxanthine (IBMX) (Sigma). Then, the cells were seeded onto 384-well plates at a density

of 7500 cells per well. The transfected cells were incubated for 45 min with gradient concentrations of formoterol at incubator. Finally, the cells were incubated with cAMP-D2 and anti-cAMP Crypate for 1 h at room temperature and time-resolved FRET signals were measured at 620 nm and 650 nm using CLARIOstar plate reader (BMG LabTech, Germany). Data were mean ± s.e.m. of three independent experiments performed in triplicate.

Cryo-EM sample preparation and data collection

An aliquot of 2.5 uL of the sample (0.5 mg/mL) was applied to plasma-treated (H_2/O_2, 10s) grids (Quantifoil R1.2/1.3 300-mesh Au Holey Carbon). The grids were blotted for 5 s at 100% humidity and 4 ℃, and then plunged into liquid ethane using a Vitrobot Mark IV (FEI).

Cryo-EM images were recorded on a Gatan K3 Summit direct electron detector in an FEI Titan Krios electron microscope at 300 kV. SerialEM was used for automated data collection[4]. Movies were collected at a nominal magnification of 29000 × in counting mode, corresponding to a pixel size of 0.53 Å. The defocus range was set to -1.5 to -2.5 μm. Image stacks were record with a total dose of ~60 e^-/Å. The electron dose rate is 20 e^-/Å/s.

Image processing

A total of 2102 micrograph stacks were collected and subjected for motion correction using motioncor2[5]. Contrast transfer function parameters were estimated with Gctf[6]. After manually removal of bad micrographs, a total of 1,781,163 particles were automatically picked from 1733 micrographs. These particles were then subjected to reference-free 2D classification and particles of the best classes were re-extracted for further data processing. A 3D initial model was generated using stochastic gradient descent (SGD) algorithm in Relion-3.0[7]. Next, 776,825

picked particles were performed for global angular searching 3D classification. 218,011 particles from the best-looking class were selected for 3D auto-refinement, which generated an EM map with an overall resolution of 4.06 Å. By post-processing and particle polishing, the final resolution of was improved to 3.8 Å. Map resolution was estimated with the gold-standard Fourier shell correction 0.143 criterion. Local resolution was estimated using Resmap[8].

Model building and refinement

The initial model for the formoterol-bound β_2AR-Gs complex was derived from BI167107-bound β_2AR-Gs complex (3SN6) followed by extensive remodeling using COOT[9]. The N-terminal residues 1-29, residues 240-264 and C-terminal residues 341-413 of β_2AR were not built due to the lack of corresponding densities. Structure refinements were carried out by PHENIX in real space with secondary structure and geometry restraints to prevent structure overfitting[10,11]. Overfitting of the model was monitored by reefing the model in one or two independent maps from the gold-standard refinement approach and testing the refined model against the other. Validation was performed in MolProbity. Figures were prepared using UCSF Chimera or PyMOL[12].

References

1. Hanson, M.A. et al. A specific cholesterol binding site is established by the 2.8 A structure of the human beta2-adrenergic receptor. *Structure* **16**, 897-905 (2008).
2. Rasmussen, S.G. et al. Structure of a nanobody-stabilized active state of the beta(2) adrenoceptor. *Nature* **469**, 175-80 (2011).
3. Norskov-Lauritsen, L., Thomsen, A.R. & Brauner-Osborne, H. G protein-coupled receptor signaling analysis using homogenous time-resolved Forster resonance energy transfer (HTRF(R)) technology. *Int J Mol Sci* **15**, 2554-72 (2014).
4. Mastronarde, D.N. SerialEM: A Program for Automated Tilt Series Acquisition on Tecnai Microscopes Using Prediction of Specimen Position. *Microsc Microanal* **9**, 1182-1183 (2003).
5. Zheng, S.Q. et al. MotionCor2: anisotropic correction of beam-induced motion for improved

cryo-electron microscopy. *Nature Methods* **14**, 331-332 (2017).
6. Zhang, K. Gctf: Real-time CTF determination and correction. *J Struct Biol* **193**, 1-12 (2016).
7. Zivanov, J. et al. New tools for automated high-resolution cryo-EM structure determination in RELION-3. *Elife* **7**(2018).
8. Kucukelbir, A., Sigworth, F.J. & Tagare, H.D. Quantifying the local resolution of cryo-EM density maps. *Nat Methods* **11**, 63-5 (2014).
9. Emsley, P., Lohkamp, B., Scott, W.G. & Cowtan, K. Features and development of Coot. *Acta Crystallogr D Biol Crystallogr* **66**, 486-501 (2010).
10. Adams, P.D. et al. PHENIX: a comprehensive Python-based system for macromolecular structure solution. *Acta Crystallogr D Biol Crystallogr* **66**, 213-21 (2010).
11. Afonine, P.V. et al. New tools for the analysis and validation of cryo-EM maps and atomic models. *Acta Crystallogr D Struct Biol* **74**, 814-840 (2018).
12. Pettersen, E.F. et al. UCSF Chimera--a visualization system for exploratory research and analysis. *J Comput Chem* **25**, 1605-12 (2004).

Supplementary Figures

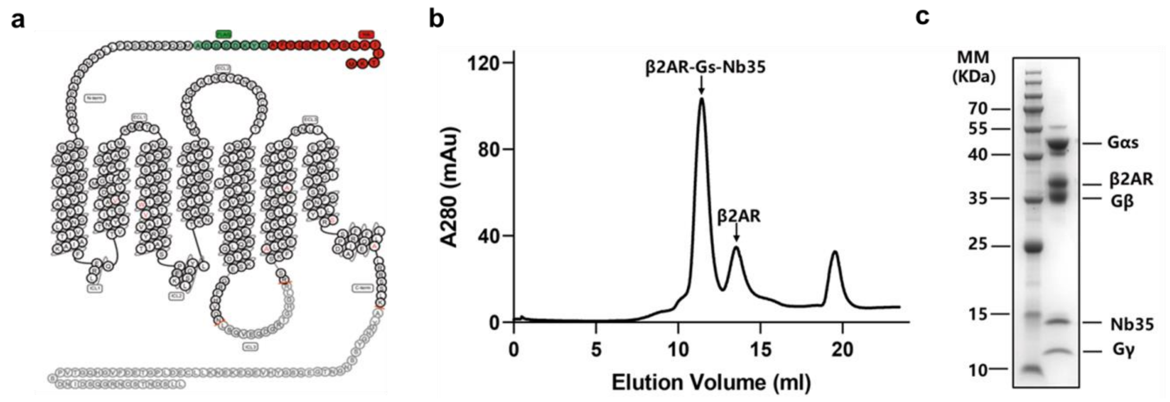

Supplementary Fig. S1 Preparation of β_2AR-Gs protein complex. a Schematic diagram of the β_2AR construct used in this study. HA signal peptide (red) and Flag epitope (green) were added in N terminus. The amino acids within ICL3 (▲245-▲259) and C terminus (▲349-▲413) were truncated to increase protein expression. **b** Size exclusion chromatography profile of formoterol-β_2AR-Gs complex. **c** SDS–PAGE analysis of final complex sample, each protein component of formoterol-β_2AR-Gs complex was clearly identified.

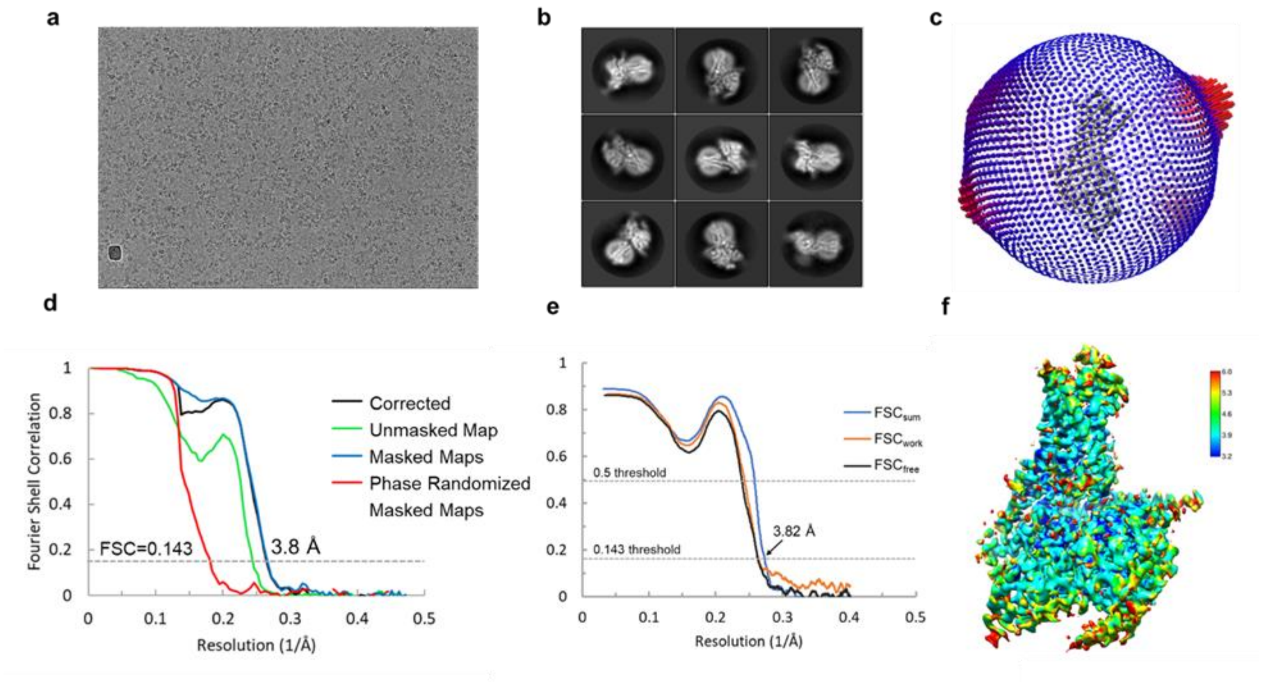

Supplementary Fig. S2 Cryo-EM analysis of formoterol-β$_2$AR-Gs complex. a Representative cryo-EM image of formoterol-β$_2$AR-Gs complex after motion correction and dose weighting. **b** Representative 2D class averages of formoterol-β$_2$AR-Gs complex. **c** Particle angular distribution of the final cryo-EM reconstruction of β2AR-Gs-Formoterol complex. **d** The gold-standard fourier shell correlation curves for the maps of formoterol-β$_2$AR-Gs complex. **e** FSCwork/FSCfree validation curves of Formoterol-β2AR-Gs complex. **f** Density map colored by local resolution estimation using Resmap.

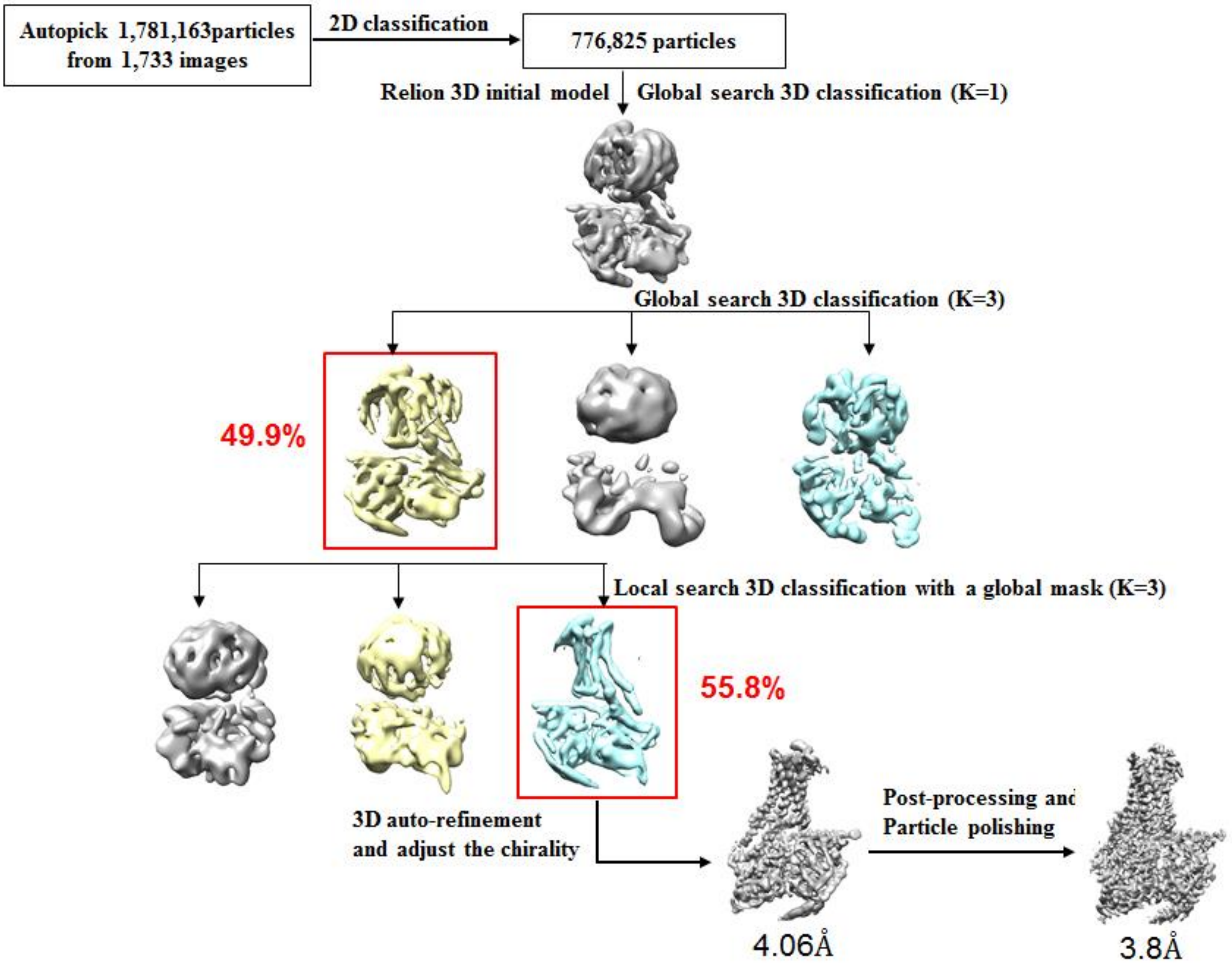

Supplementary Fig. S3 A flowchart of cryo-EM data processing and structure determination of the formoterol-β2AR-Gs complex .

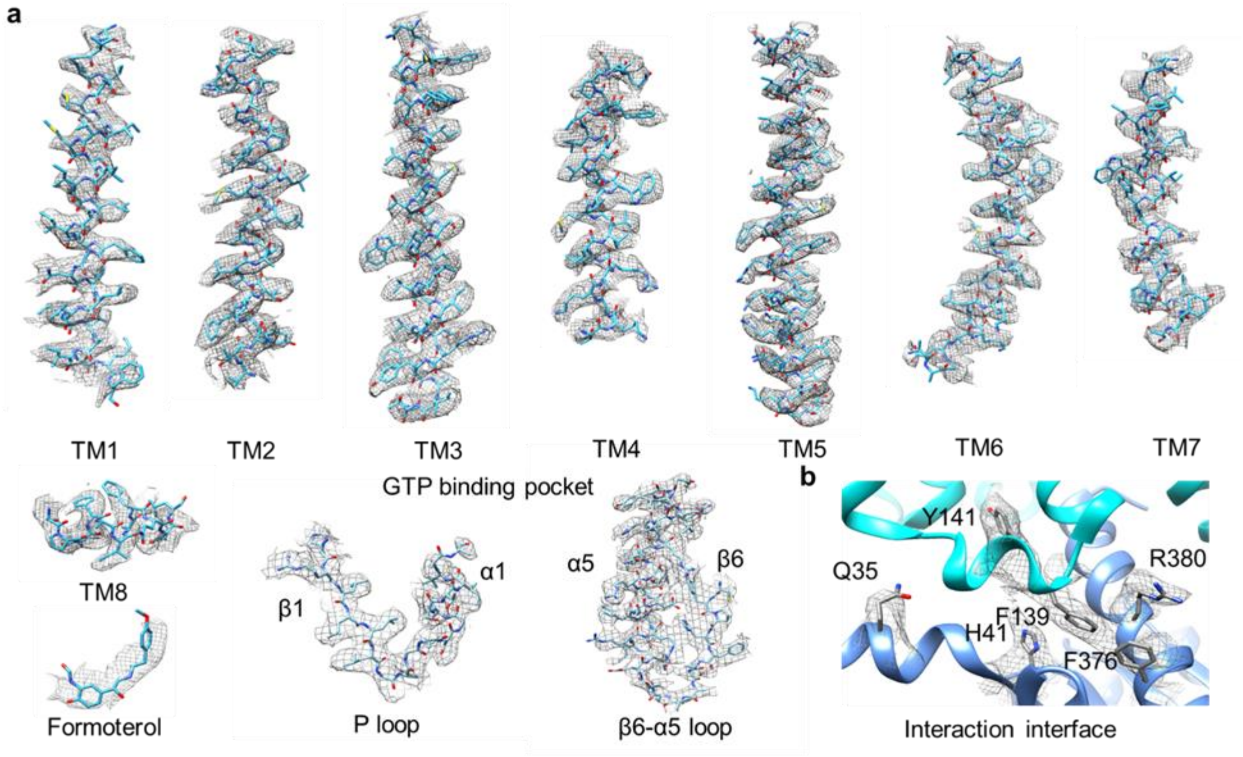

Supplementary Fig. S4 Cryo-EM densities of representative segments of human formoterol-β_2AR-Gs complex. a EM densities of representative segments of formoterol-β_2AR-Gs complex. **b** The side chain densities of residues engaged in receptor-Gs interaction interface.

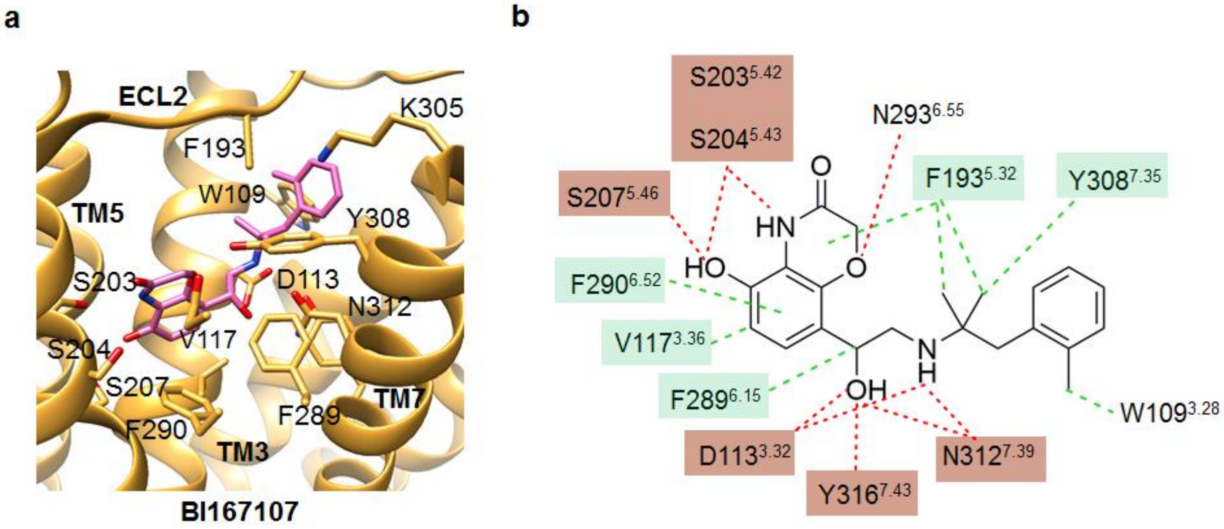

Supplementary Fig. S5 Schematic representation of the interactions between the β_2AR and the ligands BI167107. **a** Side views of ligand binding pockets in the BI167107-bound β_2AR structure. Residues within 4 Å of ligand is shown as sticks. **b** The residues shown here have at least one atom with 4 Å of the ligand in the crystal structure. Green lines indicate potential hydrophobic interactions and red lines indicate potential polar interactions.

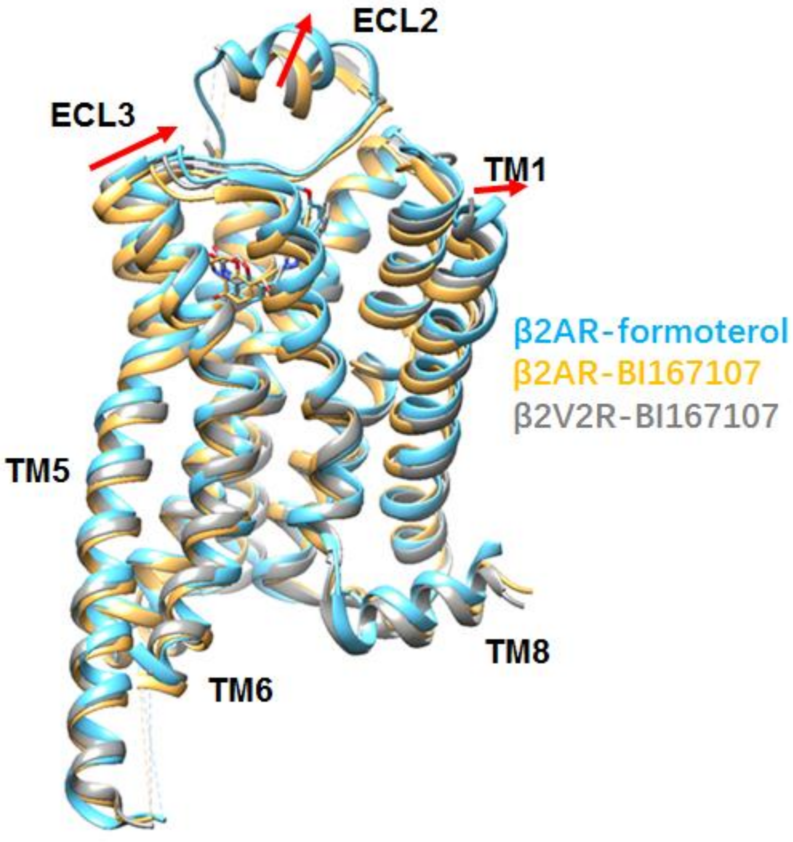

Supplementary Fig. S6 The structure comparison of the formoterol bound β_2AR, BI167107 bound β_2AR and β_2V_2R. The structures of BI167107-bound β2AR was solved by crystallography while the formoterol-bound β2AR and BI167107-bound β2V2R were solved by cryo-EM.

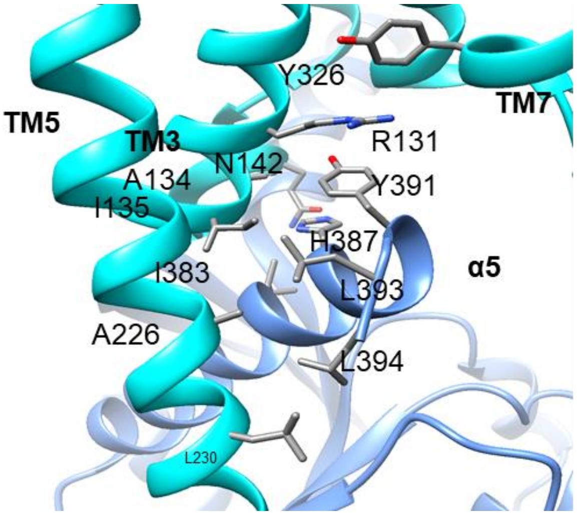

Supplementary Fig.S7 The β2AR-Gαs interface in formoterol-β2AR-Gs complex.

The α5-helix of Gαs docks into a cavity formed by the intracellular part of transmembrane helix 5 and helix 6.

Supplementary Table S1.
Statistics of cryo-EM data collection, 3D reconstruction and model refinement.

**Data collection and processing**	
Protein	formoterol-β_2AR-Gs complex
Microscope	FEI Titan Krios
Voltage (kV)	300
Detector	Gatan K3 Summit
Detector mode	Counting
Pixel size (Å)	0.53
Defocus range (μm)	-1.5~-2.5
Electron dose (e^-/ Å^2)	58.7
Magnification	29000
Number of images	1625
Symmetry imposed	C1
Initial particle images(no.)	1,781,163
Final particle images (no.)	219,254
Map resolution (Å)	3.82
FSC threshold	0.143
Map resolution range(Å)	1.06-999
**Model refinement**	
Model resolution (Å)	3.82
FSC threshold	0.143
Model composition	
Chains	6
Non-hydrogen atoms	7,393
Protein residues	978
Ligands	H98
R.m.s. deviations	
Bond length (Å)	0.004

Bond angles (°)	0.649
Validation	
MolProbity score	2.16
Clashscore	12.28
Ramachandran plot (%)	
Outliers	0.00
Allowed	10.21
Favored	89.79
